# The leading-edge vortex of swift wing-shaped delta wings

**DOI:** 10.1098/rsos.170077

**Published:** 2017-08-23

**Authors:** Rowan Eveline Muir, Abel Arredondo-Galeana, Ignazio Maria Viola

**Affiliations:** Institute for Energy Systems, School of Engineering, University of Edinburgh, Edinburgh, UK

**Keywords:** delta wing, bird wing aerodynamics, common swift, leading-edge vortex, swept wing, particle image velocimetry

## Abstract

Recent investigations on the aerodynamics of natural fliers have illuminated the significance of the leading-edge vortex (LEV) for lift generation in a variety of flight conditions. A well-documented example of an LEV is that generated by aircraft with highly swept, delta-shaped wings. While the wing aerodynamics of a manoeuvring aircraft, a bird gliding and a bird in flapping flight vary significantly, it is believed that this existing knowledge can serve to add understanding to the complex aerodynamics of natural fliers. In this investigation, a model non-slender delta-shaped wing with a sharp leading edge is tested at low Reynolds number, along with a delta wing of the same design, but with a modified trailing edge inspired by the wing of a common swift *Apus apus*. The effect of the tapering swift wing on LEV development and stability is compared with the flow structure over the unmodified delta wing model through particle image velocimetry. For the first time, a leading-edge vortex system consisting of a dual or triple LEV is recorded on a swift wing-shaped delta wing, where such a system is found across all tested conditions. It is shown that the spanwise location of LEV breakdown is governed by the local chord rather than Reynolds number or angle of attack. These findings suggest that the trailing-edge geometry of the swift wing alone does not prevent the common swift from generating an LEV system comparable with that of a delta-shaped wing.

## 1. Introduction

The leading-edge vortex (LEV) is a commonly found mechanism that, under the correct conditions, can significantly augment the lift generation of both manufactured and natural fliers [1–4]. The LEV is robust to kinematic change [1] and has been identified across a wide range of Reynolds numbers (*Re*) (table 1), from the laminar flow conditions (10<*Re*<10^4^) of autorotating seed pods [5] and in insect [1], bat [6] and small bird [7] flight, to the transitional and turbulent conditions over larger bird wings [8], fish fins [9], delta wings [10,11], helicopter rotors [12], sailing yachts [13] and wind turbines [14]. Increasingly driven by the potential exploitation of this effective lift mechanism for micro air vehicles (MAV), and facilitated by improvements in flow visualization and computational techniques, aerodynamicists and biologists alike now seek further understanding of aircraft and natural fliers at low to medium *Re* (10<*Re*<10^5^), as the LEV is increasingly understood to be a valuable flight mechanism to apply by design.

**Table 1.** Non-dimensional quantities are made non-dimensional using *l*, *U*_0_ and the density of the water.

AR	aspect ratio (*b*^2^/*S*)
*b*	span (m)
*C*_*l*_	lift coefficient
*c*_*r*_	root chord of the swift wing-shaped delta wing (m)
*l*	chordwise length of the wing (m)
*N*	number of grid points within the domain *Π*
P and M	two grid points within *Π*
*Q*	second invariant of the non-dimensional velocity-gradient tensor
*Re*	Reynolds number
*S*	area of the wing (m^2^)
*U*_0_	free stream velocity (m s^−1^)
*x*	chordwise coordinate (m)
*α*	angle of attack (deg.)
*Γ*_2_	vortex identification criterion (equation (2.1))
*Λ*	sweep back angle (deg.)
*ω*	non-dimensional vorticity
*Π*	two-dimensional, rectangular domain around P
**U**_**M**_	velocity vector at the grid point M (m s^−1^ ,m s^−1^, m s^−1^)
${\tilde{\text{U}}}_{\text{P}}$	mean velocity vector within *Π* (m s^−1^, m s^−1^, m s^−1^)
$\hat{\text{z}}$	unit vector normal to the measured plane (m, m, m)

Across the broad spectrum of delta-wing aircraft and natural fliers, an LEV’s stability and ability to augment lift depend on numerous variables. Certain variables such as wing shape, sweep back angle (*Λ*) and angle of attack (*α*) can be identified and replicated, providing the potential for comparison between the two types of fliers. The determination and modelling of the wing kinematics of a natural flier, however, poses a more complex problem than that of man-made aircraft. Vortex lift is recorded at the same *α*, 30°, on both the extensively studied slender delta wing (e.g. [15], where *Λ*=75°) and the more complex case of a bird wing, where a blunt-nosed, flapping model wing is tested at *Λ*=10° [8]. The number of variables in the latter study makes a comparison challenging, however, the simpler act of gliding flight can more readily be studied via the existing relative wealth of delta wing research; such a comparison may allow the examination of key variables in isolation.

Research into the aerodynamics of non-slender or ‘low sweep’ (*Λ*<60°) delta wings is more limited than that available on slender (*Λ*≥60°) delta wings, despite the increased relevance of the non-slender configuration, as research into MAVs becomes increasingly active [16]. Many (e.g. [16–20]) have experimentally and computationally explored non-slender delta wings of *Λ*=50°, with varying leading-edge radii and at a range of *Re* and *α*. These delta wings have a wing configuration comparable with that of the impressive natural fliers, the common swift *Apus apus*, when in non-diving, gliding flight. With a ‘hand wing’ sweep of *Λ*=50° [7], the narrow, protruding, anterior vein of the primary feather provides a sharp leading-edge [21], as is typical in delta wing design to promote leading-edge separation. The trailing-edge shape and surface area of a swift wing clearly differs from a delta-shaped wing, as the wing tapers to a point from the body to the tip. The wing shape and configuration during non-diving gliding therefore conveniently allows comparison with a normal delta-shaped wing; the tapered wing of the swift can be simply represented by a modified trailing edge, and its effect on the flow structure can thus be elucidated.

This fundamental difference in trailing-edge shape should be explored if an analogy between the intricate LEV system of a natural flier and that of a more readily examined delta wing is to be made (e.g. [22]); such an analogy has in fact been identified as critical in enhancing understanding of natural flight (e.g. [23]). The lift produced by bird wings can be described by the circulation around a streamlined body, supported in specific circumstances by the circulation of the LEV. The delta wing provides a paradigm example of the latter, where the wing lift can be almost entirely due to LEV circulation. The formation and stability of the LEV on natural fliers can therefore be studied with this simplified geometry; the absence of more complex bird wing geometry, kinematics, flexibility, etc., facilitating understanding of the underlying aerodynamic mechanisms, as the accurate testing of natural fliers continues to challenge researchers who seek to apply this robust, high-lift mechanism by design.

Taylor *et al.* [16] tested a sharp leading-edge delta wing model at *Λ*=50° and low *Re* in a water tunnel. Using particle imaging velocimetry (PIV) and ink injection, they present the first experimental evidence of the development, not of a single LEV, but a dual LEV, from 2.5°≤*α*≤15° at *Re*=13 000. A dual LEV is a system now commonly described on non-slender delta wings at low incidence [24], though, to date, it is not known to have been identified on bird wings. It comprises a larger, primary leading-edge vortex along with a second, minor, co-rotating vortex, separated by a region of counter-rotating vorticity generated between the vortices and the wing suction surface. They further show that, at *α*=7.5°, the dual LEV is recorded across a range of low to moderate *Re*, from 8700 to 34 700. Interestingly, at *Re*=26 700 neither a dual nor single LEV is present at a lower *α* of 5°, suggesting a strong sensitivity to *Re*. In contrast to this finding, Gordnier & Visbal [17] use a sixth-order compact differencing scheme to computationally reveal the existence of a dual LEV at *α*=5°, at the same *Λ* of 50°, at *Re*=20 000,26 000 and 50 000. In further contradiction to Taylor *et al.* [16], Gordnier & Visbal [17] reveal a classic primary, secondary and tertiary vortex system, more typical to a slender delta wing, at *α*=15°. At this *α* the flow becomes unsteady and vortex breakdown is seen. They corroborate the observation of Taylor *et al.* [16], that a dual LEV exists at 26 000 and *α*=10.

The experimental results of Videler *et al.* [21], who test model wings of a common swift at a characteristic slender delta wing sweep *Λ*=60°, show that a model swift wing can also generate an LEV in ‘gliding flight’. They use PIV to record flow separation, as would be expected on a slender delta wing under the same conditions, where a single LEV is noted at low *α* (5°–10°). It is asserted that while the ‘arm wing’ generates lift conventionally, being similar in cross-sectional shape to a conventional aerofoil, the hand wing section is used to generate lift via an LEV. The generation of a single rather than a dual LEV is likely to be a result of the increased sweep; however, the additional effect of the tapering of the swift wing on the flow is not known.

No force measurements are undertaken by Videler *et al.* [21], and their assertion that the LEV is used by the swift for lift augmentation is challenged by Lentink *et al.* [7]. They test real, inherently flexible, swift wings with a maximum sweep of 50°; a more representative wing position during normal, non-diving gliding. They too find a single LEV over the swift wing, but only at higher *α*. When *Re*=25 000, an LEV is only identified where *α*≥11°, a result in contrast to the delta wing findings presented earlier in this section; Taylor *et al.* [16] reveal a dual LEV at *α*=7.5° from *Re*=8700 to 34 700, and Gordnier & Visbal [17] find a dual LEV at *α*=5° when *Re*=26 000. Lentink *et al.* [7] also test the swift wings at *Re*=50 000 and only find an LEV where *α*≥14°, which again contrasts with the results of Gordnier & Visbal [17] who test at the same *Re*, and present a dual LEV at *α*=5°. In the research presented on both fliers, the existence and form of the LEV depends on factors not yet understood. A systematic approach is therefore required to understand the influence, on the flow, of the numerous variables at play.

To isolate the effect of trailing-edge geometry, which provides an approximation of the fundamental difference in wing shape, we perform flow measurements at *Re* typical of the gliding swift, between 12 000 and 67 000 [25], over two geometrically equivalent model delta-shaped wings. One of the wings was modified to crudely mimic a swift wing by removing material from the trailing edge, providing a tapering wing from the apex to the tip. The form and evolution of the LEV across a range of *α* and *Re* are revealed on both planforms using PIV. The aim of this investigation is to identify whether a swift wing-shaped delta wing can generate a comparable LEV system to that of a normal delta wing, to explore the effect of the trailing-edge shape of the swift wing on LEV generation and stability. The results will also be discussed in relation to known swift flight aerodynamics.

## 2. Material and methods

### 2.1 Model specification and facility

**Figure 1.**
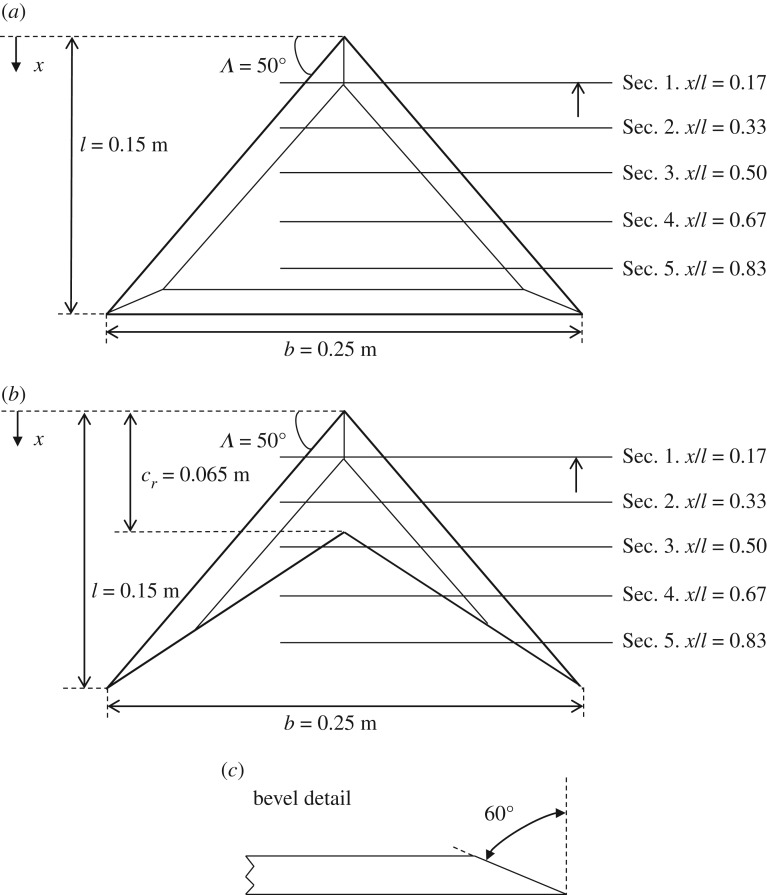
Schematic diagram of the tested models, showing the imaged sectional cross-flow planes at different chordwise coordinates *x*/*l*. (*a*) delta-shaped wing, (*b*) swift wing-shaped delta wing and (*c*) bevel detail.

The tested delta wing (figure 1) has *Λ*=50°, chordwise length *l*=0.15 *m* and span *b*=0.25 *m*, which with area *S* gives the wing an aspect ratio (*AR*=*b*^2^/*S*) of 3.36. The delta wing design was then replicated and, using the trailing-edge taper and AR provided in Lentink *et al.* [7], for a swift wing at 50° sweep, modified to produce a simple, rigid, swift wing-shaped delta wing planform. The wing shape is constructed with *Λ*=50°, root chord *c*_*r*_=0.43 *l*, a span of 0.25 and an aspect ratio of 6.

The wing sections were laser cut from 10 mm thick acrylic. A sharp leading edge is provided on both wings by applying a 60° bevel on the windward side, as is common practice in delta wing investigations (e.g. [18,26]). The leading-edge bevel also results in the wing gaining an effective camber [10,27]. Camber can, however, be seen on the cross section of bird wings (e.g. [21]), and as aerodynamic forces are not explored in this instance, this was not thought to be unduly detrimental to the study. Both models were painted matt black to reduce reflection of the laser.

**Figure 2.**
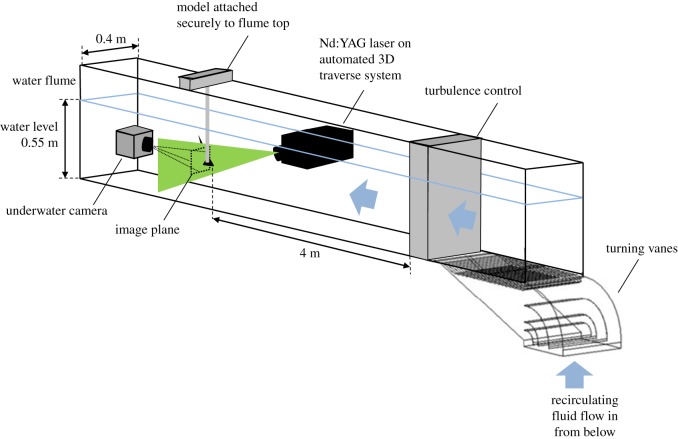
Schematic diagram of the experimental rig. The figure shows the general position of the laser, the model and the downstream location of the underwater camera.

**Figure 3.**
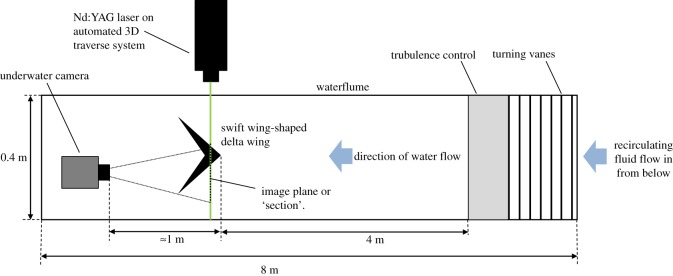
Plan view schematic of the experimental rig.

The wing was rigidly located in a low-speed, free surface, water flume at the University of Edinburgh (figures 2 and 3). The glass-walled flume is 8 m long and 0.4 m wide with a water depth of 0.55 m; it is of closed circuit, recirculating current design, and includes a series of meshes around 4 m upstream of the model to reduce turbulence. The maximum blockage ratio (the ratio between the projected area of the model and the cross-sectional area of the volume of flowing water) was lower than 3.5% for the delta wing at *α*=15°, and thus no corrections were made. While there was a relatively small clearance of 0.5 *l* between the wing tips and the flume wall, any consequence of this would have a parallel effect on both models, so no notable impact on the comparison between the two was anticipated. The water speed is controlled by an electric motor driving a propeller providing a flow speed (*U*_0_) at the model location of up to 1 m s^−1^. In the present investigation, 0.10 m s^−1^<*U*_0_<0.44 m s^−1^, resulting in a chord-based *Re* range of around 15 800 to 65 500, with the turbulence intensity ranging from 6.8% to 3.1%, respectively. Where possible, the test section of the flume was covered externally in thick matt black fabric to reduce reflection.

The models were supported by a frame securely mounted on the top of the flume, and via a 12 mm diameter cylindrical sting attached to the upper, bevelled surface of the wing (figures 2 and 3). The wing was attached with a screw countersunk into, and flush with, the suction surface; the suction surface was therefore the lower surface of the wing. The sting was attached to the frame via an adjustable bracket that allowed the angle of attack of the wing to be modified. The angle was measured and set by a digital level with an accuracy of ±0.01°.

A set of images were taken of the cross-sectional flow field over the swift wing-shaped delta wing at five different sections, namely Sec. 1 to Sec. 5 in figure 1. These sections are at a distance *x*/*l* from the apex of 0.17, 0.33, 0.50, 0.67 and 0.83, where *x* is a chordwise coordinate from the apex of the wing, and *l* is the chordwise length of the wing. Sec. 2 was selected to be sufficiently far from the apex to allow development of the flow feature, however, upstream of vortex breakdown in most cases.

The flow field was recorded at the angle at which a vortex was identified by Lentink *et al*. [7], *α*=15°; then again at steps reducing the angle of attack to *α*=0°. *α*=0°, 5°, 10° and 15° are presented. A set of images was also taken at *α*=7.5° on both the normal and swift wing-shaped delta wings, a previously documented flow regime on non-slender delta wings, to allow comparison with previously published literature (e.g. [16]). The first and second set of images were taken across the range of *Re*; however, only the lowest (*Re*=15 800) and highest (*Re*=65 500) cases are presented because the flow field was found to vary virtually linearly with *Re*. A third set of images were again taken at *α*=7.5°, but at all five locations along the length of each wing at *Re*=17 500, to elucidate vortex development along the leading edge of each planform more fully.

### 2.2 Particle imaging velocimetry

A dual-pulse Nd:YAG laser (15–200 mJ at 532 nm, 200 Hz) was used to generate a 3 mm thick light sheet illuminating seeding particles in selected planes at each *α*. The seeding particles used are Conduct-O-Fil silvered spheres, with an average diameter of 14 μm and average density of 1.7 g cc^−1^. A LaVision Imager pro SX 5M camera fitted in a waterproof housing was secured in the flume around 1 m downstream from the wing support rig. In order to improve resolution, only one half of each model was imaged. All images and raw data are available on the Edinburgh DataShare repository (http://datashare.is.ed.ac.uk).

### 2.3 Image processing and vortex detection

Analysis was undertaken using the LaVision software, DaVis 8.3.0. The images were preprocessed using image subtraction, subtracting the average image of each sample set, in order to reduce light reflections and increase particle definition close to the wing surface [28]. The preprocessed images (field of view of around 150×125 *mm*) were then broken into interrogation windows, and the wing model masked from the image and analysed using a multipass cross-correlation algorithm.

An FFT algorithm was applied with a window size of 64 pix × 64 pix and a 50% overlap, and refined with three passes at 32 pix × 32 pix window size and a 75% overlap. Outliers were removed in post-processing with a median filter, and a 3×3 Gaussian smoothing was applied. Results presented are averages of 100 image pairs taken at a frequency of 7.5 Hz, with a resolution of 2448 pix × 2050 pix. Velocity vectors and vorticity contours are presented; the vorticity field is computed from the curl of the velocity field, and only 1 in 4 velocity vectors are displayed, for clarity.

Two vortex identification algorithms were used to further elucidate the flow field. Vortical structures are identified using the *Q* criterion [29], where *Q* is the second invariant of the velocity gradient tensor. A positive value of *Q* results when the local magnitude of rotation dominates that of strain. Vortex boundaries were also computed using the *Γ*_2_ criterion as defined by Graftieaux *et al.* [30], which derives a solution not from the magnitude of the velocity field, but from the topology. For every point P, *Γ*_2_ is defined as follows:

2.1 $$\Gamma_{2}(P)=\frac{1}{N}\sum_{\Pi }\frac{[\text{PM}\wedge ({\text{U}}_{\text{M}}-{\tilde{\text{U}}}_{\text{P}})]\cdot \hat{\text{z}}}{∥\text{PM}∥\cdot ∥{\text{U}}_{\text{M}}-{\tilde{\text{U}}}_{\text{P}}∥},$$

where *Π* is a rectangular, two-dimensional domain around P. The point M, identified by the position vector **PM**, is one of the *N* points in *Π*. At the point M, **U**_**M**_ is the measured velocity and $\hat{\text{z}}$ is the unit vector normal to the measured plane. The mean convection velocity ${\tilde{\text{U}}}_{\text{P}}$ within *Π* is subtracted from **U**_**M**_ to make the algorithm Galileian invariant, as is the *Q* criterion.

## 3. Results

### 3.1 Primary and secondary leading-edge vortex

**Figure 4.**
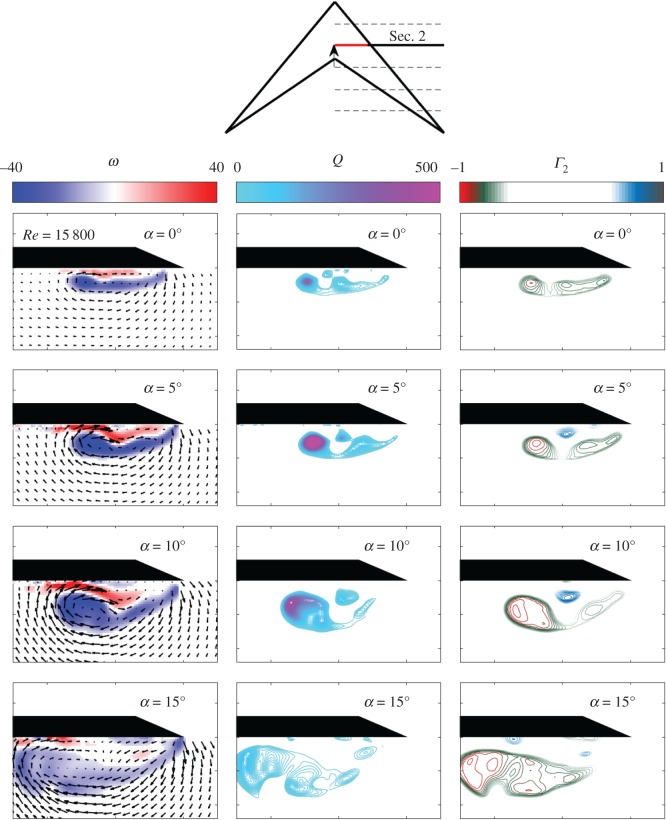
In-plane mean velocity vectors and vorticity contours, *Q* and *Γ*_2_ around the swift wing-shaped delta wing at *Re*=15 800, for a range of *α*.

**Figure 5.**
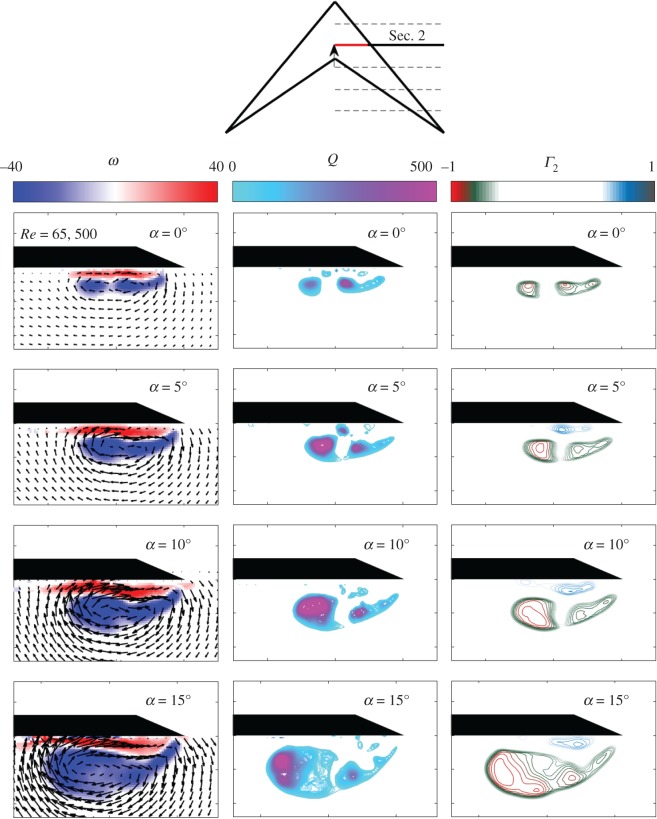
In-plane mean velocity vectors and vorticity contours, *Q* and *Γ*_2_ around the swift wing-shaped delta wing at *Re*=65 500, for a range of *α*.

 Figure 4 shows the flow field on Sec. 2 (*x*/*l*=0.33) of the swift wing-shaped delta wing at *α*=0°, 5°, 10°, 15° and *Re*=15 800. The time-averaged, in-plane, vector velocity field is presented together with the vorticity contours, the *Q* criterion and the *Γ*_2_ criterion. Figure 5 shows the same but for *Re*=65 500. At *Re*=15 800, a dual vortex system comprising a primary and second, minor LEV, along with a counter-rotating secondary vortex, is recorded in all cases tested, until vortex breakdown is imaged at *α*=15°. Breakdown was also revealed by the increased unsteadiness of the flow field. As reported previously (e.g. [10,31,32]), the LEV system increases in size and strength with increased *α*, resulting in a reduced proximity to the leading edge. In each case presented, the minor vortex (nearer to the leading edge) contains notably reduced vorticity than the primary vortex (inbound on the wing). At *α*=10°, the minor vortex is significantly weaker, suggesting breakdown. That breakdown may affect the minor vortex in advance of the primary vortex is also reported by Ol & Gharib [18], and Taylor *et al.* [16].

At *Re*=65 500 (figure 7), the same primary and second, minor dual vortex system is found at low *α* (0° and 5°). The minor vortex is now more similar in strength to the primary vortex. At higher *α* (10° and 15°), the vortex system appears to display a tertiary vortex as is reported by Gordnier & Visbal [17] at *Re*=26 000 and *α*=15°. By *α*=15°, the system shows notable unsteadiness as the breakdown moves upstream with increase in *α*. The area of secondary separation is less distinct than at *Re*=15 800; however, this could be a function of reduced resolution at the surface of the wing with slight movement of the wing due to the increased flow rate and related hydrodynamic forces. In line with Taylor *et al.* [16], the increase in *Re* also results in a slight movement of the vortex system outboard, towards the leading edge, as the effects of viscosity are reduced. The proximity of the system to the wing surface is largely unchanged.

### 3.2 Comparison between delta wing and swift wing-shaped delta wing vortex structures

**Figure 6.**
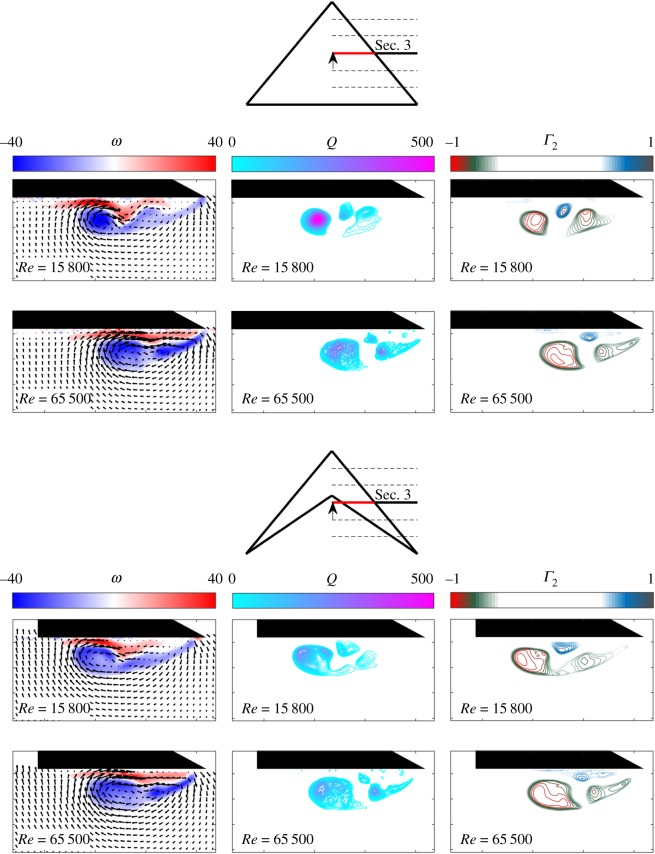
In-plane mean velocity vectors and vorticity contours, *Q* and *Γ*_2_ around the delta- and the swift-shaped wing. Tests performed at *α*=7.5° and *Re*= 15 800 and 65 500.

 Figure 6 shows that under the same hydrodynamic conditions, the flow around the delta wing and the swift wing-shaped delta wing is highly comparable, both developing a coherent dual vortex flow structure located around the same horizontal and vertical position over the suction surface of the wing. In both cases, the primary LEV and second, minor, co-rotating vortex can be seen clearly in the *Q* and *Γ*_2_ vorticity contour plots, along with the distinct secondary counter-rotating vortex. The primary difference appears to be the increased size, but reduced coherence and strength, of the swift wing-shaped delta wing vortex structure compared with that of the delta wing. This is particularly apparent at the lower *Re*. The strength and magnitude of the secondary vortex in the swift wing-shaped case is also reduced, again most notably for *Re*=15 800. At higher *Re*, the difference between the two systems is reduced but still may be perceived. As previously described for figures 4 and 5, the vortex structures shift position outboard slightly as expected [16] with increase in *Re*.

### 3.3 Vortex breakdown

**Figure 7.**
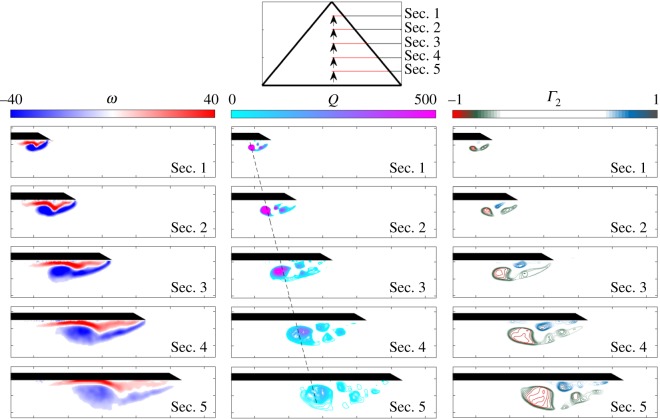
In-plane mean velocity vectors and vorticity contours, *Q* and *Γ*_2_ around the delta-shaped wing. Tests performed at *Re* = 17 500 and *α*=7.5°.

**Figure 8.**
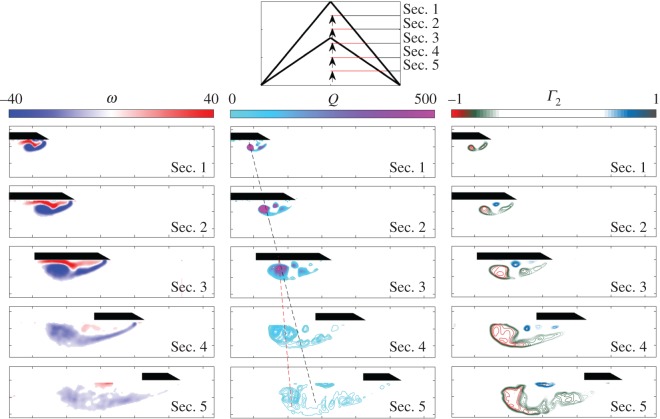
In-plane mean velocity vectors and vorticity contours, *Q* and *Γ*_2_ around the swift wing-shaped delta wing. Tests performed at *Re* = 17 500 and *α*=7.5°.

Figures 7 and 8 show the vortex development along the span of normal and the swift wing-shaped delta wings, respectively, at *α*=7.5° and *Re*=17 500. While the flow field towards the root of the wing models is highly comparable in vorticity magnitude, location and coherence, differences are seen from around Sec. 3 (*x*/*l*=0.5). In the case of the swift wing-shaped delta wing, the vortex system appears to suffer the loss of coherence and increase in size associated with breakdown, as the width of the vortex system exceeds that of the local wing chord beneath it, and flow reattachment is no longer possible. This transition is seen clearly by reviewing Sec. 3 and Sec. 4 on both models; while the vortex system of the delta wing (figure 7) at Sec. 3 continues to be supported by the delta wing surface in Sec. 4, the tapering swift wing (figure 8) no longer supports the necessary flow reattachment and at Sec. 4, vortex breakdown is seen. Following breakdown, a larger region of vorticity made of small-scale, unsteady vortical structures is seen, with a concentration in the region of the primary LEV. The delta wing vortex system, on the other hand, largely retains coherence. The trajectory of the delta wing primary vortex is tracked, showing the location of the vortex along the wing. The trajectory of the swift wing-shaped primary vortex is also tracked; however, it can be seen from Sec. 4 that the vortex deviates notably from its upstream trajectory. In line with the experimental results of Ol & Gharib [18], and Taylor *et al.* [16], the second minor vortex displays the greatest reduction in coherence; it is proposed that this may serve to provide an early indication of LEV breakdown.

## 4. Discussion

The trailing edge of a delta wing was modified to produce a swift wing-shaped delta wing. The figures presented show that the modification does not intrinsically prevent the development of a leading-edge vortex system under typical gliding conditions of a swift, at low *α* and *Re*, that is highly comparable to that of a classic delta wing.

Figures 4 and 5 present the averaged flow field towards the apex of the modified, swift wing-shaped delta wing. In line with the computational study of non-slender delta wings at *Λ*=50° and *α*=5°,10° and 15° [17], a shallow separated shear layer can be seen at low *α*, which increases in prominence and strength with increase in *α*. The flow separates from the sharp leading edge forming a shear layer which rolls up into a coherent LEV. At lower *Re*, secondary separation of opposite sign can be seen between the LEV and the wing surface, formed by the interaction between the LEV and the boundary layer (e.g. [10,16,18]). Dual vortices are more readily developed on non-slender rather than slender delta wings due to the increased proximity of the LEV to the wing suction surface with reduced sweep [10]. At *Re*=15 800, the minor vortex is not clearly defined by vorticity contours alone, as it is almost entirely embedded in the shear layer (in line with [17]); however, isolines of positive *Q* and *Γ*_2_ show three distinct vortices until breakdown at *α*=15°. Vortex breakdown progresses upstream with increase in *α* (e.g. [18]); as seen in similar investigations on delta wings (e.g. [16]), the broken-down vortex remains located over the wing, with the vertical position of the vortex axis largely unaffected.

The primary, secondary and tertiary co-rotating vortex system identified at *α*=10° and *α*=15° at *Re*=65 500 is an interesting observation when compared with Gordnier & Visbal [17]. They find this system only where *α*=15° at *Re*=26 000, so their assertion that the vortex system on non-slender wings (as opposed to that of slender wings) is sensitive to changing *Re* would appear to be supported. In this case, it may be that increase in *Re* promotes the development of the tertiary structure at a lower *α*.

 Figure 6 allows a comparison between the vortex system on the delta wing and the swift-shaped delta wing, where perhaps the most noteworthy outcome is their similarity in form, particularly at lower *Re*. The vortex of the swift wing-shaped wing suffers greater disturbance through advanced breakdown; however, this does not seem to notably impact the form and location of the vortex system upstream of the point of breakdown. This suggests that an analogy between the vortex systems on a delta wing and a more realistic swift wing may not fail due to the wing trailing-edge shape alone. The noted reduction in coherence of the swift wing-shaped delta wing vortex system is typically associated with reduced lift, while the increase in size increases drag.

The relationship between the spanwise position of vortex breakdown and the local chord length of the wing is elucidated in figures 7 and 8. While the swift-wing shape does not in itself prevent the generation of a vortex system, it does appear to provide a physical limit that is not experienced by a delta wing. This limit provides a maximum LEV size, after which point any increase in *α* or advance ratio, for example, would result in vortex breakdown, reducing lift and increasing drag. Breakdown can therefore be mediated by controlling the width of the vortex system; an increase in *Re*, for example, has been shown to increase coherence and shift the vortex system outboard, thereby delaying the width of the system from reaching that of the local chord length. The proposed relationship between vortex size and wing chord is aligned with the previous work of Rival *et al.* [33], who find that, for plunging profiles, with varying leading-edge geometries, vortex detachment occurs at an LEV length scale of one chord length. While the vortex system does not detach in this study, the point of breakdown is governed by the spanwise location at which the width of the LEV system reaches one local chord length.

The identification of a dual LEV at low *Re* is in contrast to both the PIV results of Videler *et al.* [21] and the tuft flow visualization of Lentink *et al.* [7], who provide the closest comparative experimentation on wings which more closely replicate natural fliers. While the PIV measurements of Videler *et al.* [21] found a prominent but single LEV on a model swift wing at *α*>5°, their model had a slender sweep of *Λ*=60°, bringing improved vortex stability. Had they tested at *Λ*=50°, as in the present work, it is likely that they would not have found an LEV at a lower *α* due to reduced LEV stability of non-slender wings at low *α*. The probable lack of a vortex at low *α*, and that no dual vortex was recorded in any case, are both in contrast to the results presented in this paper. Similarly, Lentink *et al.* [7] did not record an LEV at low angles of attack, when they tested real swift wings at *Λ* = 50°, finding evidence only at *α*≤11°. They also did not record a dual vortex in any case.

These results suggest that the increase in *α* required to generate an LEV on the swift wings tested previously is not due to the wing trailing-edge geometry, but to another variable. It is easily conceivable that the differences in results noted here are due to varying sharpness of the leading edge or wing thickness across the respective models tested. In the case of the dual LEV, the surface texture or structure of the bird wing could affect the nature of the secondary separation and its influence on the vortex structure.

It may also be that the flow visualization method used is key in identifying the smaller, weaker vortex structure at low *α* and *Λ*. Videler *et al.* [21] do not state the resolution of their PIV analysis, however their use of a thick laser sheet of 30 mm (on a hand wing section of average chord length 50 mm) may have prevented any finer flow features from being resolved. Lentink *et al.* [7] use a tuft grid survey, originally developed for testing flow over slow-flying delta wings [34]. The tuft grid method places a tuft (or single hair) at regular intervals across the wing which can then be imaged as it moves according to the local flow regime. Where the size, strength, position etc. of the flow feature is not known, it can be difficult to suitably define the length, stiffness and position of the tuft to highlight the sought feature. It may be possible that a weak LEV did exist in the cases presented, but that the flow visualization technique used was not sensitive enough to capture it. Similarly, a dual vortex may have existed at higher angles of attack with the weaker minor vortex not identified for the same reason.

The present results on the flow structure over a tapering swift wing-shaped delta wing allow speculation on the flow structure over a real swift wing, and its potential use. The effect of the swift wing geometry is exaggerated in the test case presented here by the more pronounced reduction in chord length of the model, compared with that of a typical swift wing planform [35]. A twisted leading edge and wing flexibility would also affect the breakdown location; a spanwise reduction in the effective angle of attack via twist or flexibility would attenuate vortex development towards the wing tip, delaying the onset of vortex breakdown, despite the tapered wing.

The many differences between the wing of a real swift, such as that tested by Lentink *et al*. [7], and the model presented here have arguably been ‘selected’ in natural fliers to improve their aerodynamic and flight ability. The leading-edge detail, in particular the sharpness, is known not only to promote flow separation, but also to define the relationship between *α* and the force coefficients generated [36], while the behaviour of the LEV is increasingly dependent on leading-edge shape at low sweep [37]. If a vortex can be generated at low *α*, promoted by the sharp leading edge of the swift wing and stabilized in part by leading-edge twist and wing flexibility, it is interesting to consider what function it may serve if not one of lift augmentation, or lift-to-drag ratio improvement. While Henningsson *et al.* [38] conclude that the swift wing is best adapted for flapping rather than gliding flight, the ability to generate and maintain a stable leading-edge vortex across a wide range of *α* may be a useful addition to the suite of flight optimization tools deftly deployed by the swift. As alluded to by their genus name *Apus* (from the Greek ‘a pous’, meaning ‘without foot’), swifts spend the majority of their lives in the air; hunting, eating, collecting water, mating and even roosting [7], and they have necessarily evolved to be one of the most aerodynamically refined bird species [25]. The optimization of energy required to undertake these necessary activities is clearly essential, and with the swift spending a significant portion of its flight time gliding, often in complex air flows, any reduction in gliding energy requirement would be of significant benefit.

While the LEV is typically referred to as a high-lift mechanism [1–5], it is known that the component of total lift provided by vortex lift reduces with decreasing sweep [39]. It may be that the ability to generate an LEV and/or LEV system on a swift wing can provide another function. Along with potentially enhancing lift and manoeuvrability at high *α* and during flapping flight, the LEV system may help to manage wing loading and aerodynamic force fluctuations at lower *α*, by acting as a dampening mechanism. The ‘robustness to kinematic change’ noted by Ellington [1] may allow the LEV to react more slowly to sudden changes in the angle of attack that might otherwise have resulted in a sharp increase in lift, or in flow separation and stall. Rather, the LEV may grow or reduce in size at a rate mediated by inertia of the vortex system, altering the lift-to-drag ratio, but preventing the severe loading fluctuations associated with stall. The ability to exploit the LEV in this way could save the bird significant expenditure of energy, and may be a useful addition to a suite of flow control techniques used by MAVs seeking to fly in complex or turbulent weather conditions.

## 5. Conclusion

The flow structure over a model delta wing and a model swift wing-shaped delta wing has been characterized, with the aim of elucidating the effect of the tapering of the swift wing on the wing’s aerodynamics. The models have identical sharp leading edges, with a non-slender sweep angle of 50°. The swift wing-shaped delta wing has a modified trailing edge, resulting in a crude tapering wing planform.

A leading-edge vortex system was found in all cases tested, from *α*=0° to 15° and from *Re*=15 800 to 65 500, on both the normal and modified delta wing models. The cross-flow planes present the mean velocity vectors and vorticity contours, along with iso-surfaces of *Q* and *Γ*_2_. At lower *α* and *Re*, a clear primary and second minor co-rotating vortex structure is present, along with an area of counter-rotating vorticity between the dual LEV and the wing surface. At higher *α* and *Re*, a tertiary co-rotating vortex is identified, as is typical on slender delta wings. The size and strength of the vortex system increases with *α*, while increased *Re* improves coherence, and results in the vortex system moving outboard.

The results presented demonstrate that the flow developed over the two-wing platforms is highly comparable, despite the tapering of the swift wing-shaped wing. The notable difference between the two is the location of vortex breakdown along the wing span. On the swift wing-shaped delta wing, this is governed by the width of the vortex system in relation to the local wing chord length; where the size of the vortex system exceeds that of the local wing chord, flow is no longer able to reattach and the vortex breaks down. The vortex system upstream of the point of breakdown is not significantly impacted in the cases presented. The broken-down vortex remains attached to the wing suction surface, with the proximity to the surface remaining largely unaltered, compared with that of the delta wing. The results presented confirm the sensitivity of the non-slender wing shapes to changing *Re*. An increase in *Re* results in increased coherence of the vortex system, and also a movement of the system towards the leading edge, both serving to reduce the overall width of the system. It is hypothesized that an increase in *Re* would also therefore delay vortex breakdown for the swift wing-shaped delta wing, as the width of the vortex system compared with the local wing chord is mediated.

A dual LEV is commonly reported over non-slender delta wings at low *α*, however, no dual vortex has been found over swift wings, despite their broadly comparable geometry. While numerous variables separate these two fliers, this study demonstrates that, at the same *α* and *Re*, relevant for swifts in gliding flight, a highly comparable vortical flow structure is generated by the model delta wing and the model swift wing-shaped delta wing. This suggests that if the flow fields reported over swift wings do differ from those of delta wings, it is due not to the tapering of the wing, but to other variables such as wing surface texture or leading-edge geometry.

## Data accessibility

The metadata underpinning these results and electronic supplementary material for this article can be found online on the Edinburgh DataShare repository: http://datashare.is.ed.ac.uk.

## References

[1] Ellington CP 1999 The novel aerodynamics of insect flight: applications to micro-air vehicles. *J. Exp. Biol.* 202, 3439–3448. 10562527 10.1242/jeb.202.23.3439

[2] Garmann DJ, Visbal MR, Orkwis PD 2013 Three-dimensional flow structure and aerodynamic loading on a revolving wing. *Phys. Fluids* 25, 034101 (doi:10.1063/1.4794753)

[3] Jardin T, David L 2014 Spanwise gradients in flow speed help stabilize leading-edge vortices on revolving wings. *Phys. Rev. E* 90, 013011 (doi:10.1103/PhysRevE.90.013011) 10.1103/PhysRevE.90.013011 25122373

[4] Srygley RB, Thomas ALR 2002 Unconventional lift-generating mechanisms in free-flying butterflies. *Nature* 420, 660–664. (doi:10.1038/nature01223) 12478291 10.1038/nature01223

[5] Lentink D, Dickson WB, van Leeuwen JL, Dickinson MH 2009 Leading-edge vortices elevate lift of autorotating plant seeds. *Science* 324, 1438–1440. (doi:10.1126/science.1174196) 19520959 10.1126/science.1174196

[6] Muijres FT, Johansson LC, Barfield R, Wolf M, Spedding GR, Hedenström A 2008 Leading-edge vortex improves lift in slow-flying bats. *Science* 319, 1250–1253. (doi:10.1126/science.1153019) 18309085 10.1126/science.1153019

[7] Lentink D, Müller UK, Stamhuis EJ, de Kat R, van Gestel W, Veldhuis LLM, van Leeuwen JL 2007 How swifts control their glide performance with morphing wings. *Nature* 446, 1082–1085. (doi:10.1038/nature05733) 17460673 10.1038/nature05733

[8] Hubel TY, Tropea C 2010 The importance of leading edge vortices under simplified flapping flight conditions at the size scale of birds. *J. Exp. Biol.* 213, 1930–1939. (doi:10.1242/jeb.040857) 20472780 10.1242/jeb.040857

[9] Borazjani I, Daghooghi M 2013 The fish tail motion forms an attached leading edge vortex. *Proc. R. Soc. B* 280, 20122071 (doi:10.1098/rspb.2012.2071) 10.1098/rspb.2012.2071 PMC3574357 23407826

[10] Gursul I, Gordnier R, Visbal M 2005 Unsteady aerodynamics of nonslender delta wings. *Prog. Aerosp. Sci.* 41, 515–557. (doi:10.1016/j.paerosci.2005.09.002)

[11] Gursul I, Wang Z, Vardaki E 2007 Review of flow control mechanisms of leading-edge vortices. *Prog. Aerosp. Sci.* 43, 246–270. (doi:10.1016/j.paerosci.2007.08.001)

[12] Corke TC, Thomas FO 2015 Dynamic stall in pitching airfoils: aerodynamic damping and compressibility effects. *Annu. Rev. Fluid Mech.* 47, 479–505. (doi:10.1146/annurev-fluid-010814-013632)

[13] Viola IM, Bartesaghi S, Van-Renterghem T, Ponzini R 2014 Detached eddy simulation of a sailing yacht. *Ocean Eng.* 90, 93–103. (doi:10.1016/j.oceaneng.2014.07.019)

[14] Larsen JW, Nielsen SRK, Krenk S 2007 Dynamic stall model for wind tubine airfoils. *J. Fluids Struct.* 23, 959–982. (doi:10.1016/j.jfluidstructs.2007.02.005)

[15] Visbal MR 1996 Computed unsteady structure of spiral vortex breakdown on delta wings. In *AIAA 27th Fluid Dynamics Conf., New Orleans, LA, 17–20 June*, paper 96-2074. Reston, VA: AIAA (doi:10.2514/6.1996-2074)

[16] Taylor GS, Schnorbus T, Gursul I 2003 An investigation of vortex flows over low sweep delta wings. In *AIAA 33rd Fluid Dynamics Conf., Orlando, FL, 23–26 June*, pp. 1–13. Reston, VA: AIAA.

[17] Gordnier RE, Visbal MR 2005 Compact difference scheme applied to simulation of low-sweep delta wing flow. *AIAA J.* 43, 1744–1752. (doi:10.2514/1.5403)

[18] Ol MV, Gharib M 2003 Leading-edge vortex structure of nonslender delta wings at low Reynolds number. *AIAA J.* 41, 16–26. (doi:10.2514/2.1930)

[19] Verhaagen NG 2011 Flow over 50° delta wings with different leading-edge radii. In *AIAA 49th Aerospace Sciences Meeting including the New Horizons Forum and Aerospace Exposition, Orlando, FL, 4–7 January*, pp. 1–14. Reston, VA: AIAA.

[20] Jin-Jun W, Wang Z, 2008 Experimental investigations on leading-edge vortex structures for flowover non-slender deltawings. *Chin. Phys. Lett.* 25, 2550–2553. (doi:10.1088/0256-307X/25/7/060)

[21] Videler JJ, Stamhuis EJ, Povel GDE 2004 Leading-edge vortex lifts swifts. *Sci. New Ser.* 306, 1960–1962. (doi:10.1126/science.1104682) 10.1126/science.1104682 15591209

[22] Ellington CP, Van Den Berg C, Willmott AP, Thomas AL 1996 Leading-edge vortices in insect flight. *Nature* 384, 626–630. (doi:10.1038/384626a0)

[23] Garmann DJ, Visbal MR 2014 Dynamics of revolving wings for various aspect ratios. *J. Fluid Mech.* 748, 932–956. (doi:10.1017/jfm.2014.212)

[24] Taylor G, Wang Z, Vardaki E, Gursul I 2007 Lift enhancement over flexible nonslender delta wings. *AIAA J.* 45, 2979–2993. (doi:10.2514/1.31308)

[25] Lentink D, de Kat R 2014 Gliding swifts attain laminar flow over rough wings. *PLoS ONE* 9, e99901 (doi:10.1371/journal.pone.0099901) 24964089 10.1371/journal.pone.0099901 PMC4070913

[26] Wang J, Zhao X, Liu W, Tu J 2007 Experimental investigation on flow structures over nonslender delta wings at low Reynolds numbers. *J. Exp. Fluid Mech.* 21, 1–7.

[27] Verhaagen NG 2012 Leading-edge radius effects on aerodynamic characteristics of 50-degree delta wings. *J. Aircr.* 49, 521–531. (doi:10.2514/1.C031550)

[28] Honkanen M, Nobach H 2005 Background extraction from double-frame PIV images. *Exp. Fluids* 38, 348–362. (doi:10.1007/s00348-004-0916-x)

[29] Hunt JCR, Wray AA, Moin P 1988 Eddies, streams and convergence zones in turbulent flows. In *Proc. of the Summer Program 1988, Report CTR-S88*, pp. 193–208. Stanford, CA: Center for Turbulence Research.

[30] Graftieaux L, Michard M, Grosjean N 2001 Combining PIV, POD and vortex identification algorithms for the study of unsteady turbulent swirling flows. *Meas. Sci. Technol.* 12, 1422–1429. (doi:10.1088/0957-0233/12/9/307)

[31] Ozen CA, Rockwell D 2011 Flow structure on a rotating plate. *Exp. Fluids* 52, 207–223. (doi:10.1007/s00348-011-1215-y)

[32] Wojcik CJ, Buchholz JHJ 2014 Parameter variation and the leading-edge vortex of a rotating flat plate. *AIAA J.* 52, 348–357. (doi:10.2514/1.J052381)

[33] Rival DE, Kriegseis J, Schaub P, Widmann A, Tropea C 2014 Characteristic length scales for vortex detachment on plunging profiles with varying leading-edge geometry. *Exp. Fluids* 55, 1660 (doi:10.1007/s00348-013-1660-x)

[34] Bird JD 1969 Tuft-grid surveys at low speeds for delta wings. NASA Technical Note D-5045.

[35] Henningsson P, Hedenström A 2011 Aerodynamics of gliding flight in common swifts. *J. Exp. Biol.* 214, 382–393. (doi:10.1242/jeb.050609) 21228197 10.1242/jeb.050609

[36] Usherwood JR, Ellington CP 2002 The aerodynamics of revolving wings I. Model hawkmoth wings. *J. Exp. Biol.* 205, 1547–1564. 12000800 10.1242/jeb.205.11.1547

[37] Miau JJ, Kuo KT, Liu WH, Hsieh SJ, Chou JH 1995 Flow developments above 50-deg sweep delta wings with different leading-edge profiles. *J. Aircr.* 32, 787–794. (doi:10.2514/3.46792)

[38] Henningsson P, Hedenström A, Bomphrey RJ 2014 Efficiency of lift production in flapping and gliding flight of swifts. *PLoS ONE* 9, e90170 (doi:10.1371/journal.pone.0090170) 24587260 10.1371/journal.pone.0090170 PMC3938594

[39] Polhamus EC 1966 A concept of the vortex lift of sharp-edge delta wings based on a leading-edge-suction analogy. National Aeronautics and Space Administration, NASA TN D-3767.

